# Treatment of an ossifying fibroma of the mandible with endodontic microsurgery and grafting with a biphasic calcium sulfate material: a case report

**DOI:** 10.1097/MS9.0000000000001068

**Published:** 2023-07-15

**Authors:** Damian Dudeck, Oliwia Warmusz, Edyta Reichman-Warmusz, Gregori M. Kurtzman

**Affiliations:** a “Artmedica” Ambulatory Oral Surgery and Implantology, Thorn; b Department of Histology and Cell Pathology in Zabrze, School of Medicine with the Division of Dentistry, Medical University of Silesia in Katowice; c University of Technology, Department of Medicine, Katowice, Poland; d Private Practice, Silver Spring, Maryland, USA

**Keywords:** apicoectomy, biphasic calcium sulfate, guided bone regeneration, odontogenic cyst, ossifying fibroma

## Abstract

A significant percentage of lesions of endodontic origin require surgical management due to the possible diagnosis of odontogenic cysts and tumors in the maxilla and mandible. Ossifying fibroma is a benign fibro-osseous lesion that typically presents as a painless, slow-growing, and expansile lesion that appears as a well-demarcated lesion with a variable degree of internal calcification on radiography. Treatment results in a large osseous defect, utilization of a graft to fill the void accelerates healing and prevents complications that may result from failure to fill by the host response.

**Case presentation:**

Following endodontic surgery placement of osseous graft material via Guided Tissue Regeneration to fill the defect aids to accelerate fill of the defect on a healthy 26-year-old female patient. A case discussing the one-step treatment of an ossifying fibroma of the anterior part of the mandible following endodontic microsurgery with associated retrograde fill of the apex, then site grating with biphasic calcium sulfate (Bond Apatite^®^) used in regeneration of the osseous defect related to the lesion and resulting surgery.

**Clinical discussion:**

Histologically, the ossifying fibroma is dominated by connective tissue containing cell rich areas with a few fragments of fibrosis. Moreover, in the connective tissue numerous small fragments of spongy and compact bone with areas of partial necrosis present and a significant number of inflammatory cells are observed. Surgical removal of the cyst with thorough curettage of the osseous walls and grafting of the defect provides predictable healing and the desired clinical results sought. Utilization of the biphasic calcium sulfate graft material allows the elimination of the need to overlay the area with a membrane before the flap due to its hard set and the prevention of soft tissue ingrowth into the graft material during the healing phase. Additionally, the hard set of the material allows tenting of the area to maintain the desired volume and ridge contour. Conversion of the graft material depending on the volume placed to host bone occurs over a 3–6 month period.

**Conclusion:**

The case report presented, as well as the authors experience mimics the literature on biphasic calcium sulfate in its use as an osseous graft material and is an effective method for the repair of osseous defects that result from the removal of tumors and cysts of the maxilla and mandible. Treatment of an ossifying fibroma is an ideal application of the use of this biphasic calcium sulfate material allowing tenting of the surgical site over the defect created after cyst removal without the need for resorbable collagen membranes. This simplifies its use and decreases material costs that may hamper patient acceptance of treatment without a decrease in expected clinical results.

## Introduction

Highlights

- A significant percentage of lesions of Endodontic origin require treatment due to the diagnosis of odontogenic cysts and benign tumors requiring surgical treatment.
- Ultrasonic devices and retrograde filling materials allow for proper apical preparation and backfilling of the canal of the resected tooth.
- Synthetic graft materials have benefits over available allograft and xenograft materials related to lower cost and have demonstrated comparable clinical results.
- Biphasic calcium sulfate is a biocompatible, osteoconductive, bioactive, and bacteriostatic material and well suited for filling of the cystic site following enucleation and retro fill of the canal system.

A significant percentage of patients presenting with lesions of endodontic origin require treatment due to the diagnosis of possible diagnosis of odontogenic cysts (OC) and tumors in the maxilla and mandible. The majority of those cysts and tumors, of various etiologies, require surgical treatment. The most common lesions identified include the germinal cyst, a broadly understood root cyst, as a chronic inflammatory pathological condition, may constitute the highest percentage of recognizable lesions of this type in the maxilla and mandible. Of those reported, 54.6% were radicular cysts, 20.6% were dentigerous cysts and 11.7% were keratocystic odontogenic tumors^1^. A recent study over a 30-year period reported among all cystic lesions, 92.9% were OC and only 7.1% were nonodontogenic cysts^2^. Providing, a strong link to those cysts being of endodontic origin.

In addition, benign tumors constitute a smaller percentage of pathological changes in the jawbones. Some of those tumors may appear like cysts radiographically, especially in the early stages of development, as observed with the ossifying fibroma. One type found dentally is the ossifying fibroma, an encapsulated benign neoplasm composed of varying amounts of bone or cementum-like tissue in the fibrous tissue stroma^3^. These are slow-growing benign neoplasms found most often in the mandible, but may also be found in the maxilla, that is composed of bone that develops within fibrous connective tissue. Ossifying fibromas are composed of bone that develops within fibrous connective tissue. Some ossifying fibromas consist of cementum-like calcifications, while others contain only bony material; however, a mixture of these calcification types is commonly seen in a single lesion. These are divided into three variants including cement-ossifying fibroma, juvenile psammomatoid ossifying fibroma, and juvenile trabecular ossifying fibroma (JTOF) as identified histologically, but are treated in a similar fashion^4^. A much more aggressive course has been reported with JTOFs, which are typically observed in patients younger than 15. These rapidly increase in size, and aggressive excision or more aggressive resection with 5 mm margins is recommended depending on if the lesion is recognized early with well-confined borders within the bone. This should not be confused with the common ossifying fibroma^5^. Ossifying fibroma’s, mainly occur between the age of 20 and 40^3^. These are more frequently located in the mandible, showing a definite female predilection^6^. Other cranial and facial bones, the periorbital, frontal, ethmoid, sphenoid, and temporal bones are also relatively common sites of this tumor^7^. Ossifying fibroma’s are a slow-growing lesion that is well-demarcated from the adjacent bone. Some of those lesions may grow to become massive, causing esthetic and functional deformities. Ossifying fibromas, are divided into three types which due to the presence of both bone and cementum-like tissue are described using the terms ossifying fibroma, cemento-ossifying fibroma, and cementifying fibroma^4,8–11^. The consensus, nonetheless is the three terms describe the same underlying type of lesion^4^. A much more aggressive course has been reported with JTOF and juvenile psammomatoid ossifying fibroma’s^12–15^. The treatment of ossifying fibroma’s is surgical enucleation.

Various methods are used in the surgical treatment of OC and benign tumors; from savers (sparing) through aggressive to extremely radical treatment approaches. In the group of sparing methods, an interesting alternative to the well-known marsupialization is a two-stage treatment, in which cyst or tumor decompression is utilized first. Interestingly, a one-step method using endodontic microsurgery techniques has been successfully utilized. The development of microsurgical techniques with the use of dedicated microtools and optical instruments, such as operating microscopes make effective treatment in the operating micro area, without the need to remove excessive amounts of osseous tissue and periodontal apparatus. Additionally, ultrasonic devices and new retrograde filling materials allow for proper apical preparation and backfilling of the canal of the resected tooth.

Following flap and visualization of the apical site when treating the cyst, a defect is present in the surrounding osseous area that was occupied by the cyst and resulted in resection of the apical portion of the root. Debate has circulated for many years on the need to fill the osseous surgical site. Some practitioners advocating allowing clot formation in the defect and surrounding host tissue to convert that clot to host bone. Other practitioners advocating placement of osseous graft material (guided bone regeneration) to fill the defect and accelerate fill of the defect with host bone over time. Although guided bone regeneration did not improve the clinical outcome, it did improve the quality of apical bone remodeling following **e**ndodontic microsurgery^16^. One study reported that the volume of the periapical lesion had a significant effect on the outcome of endodontic microsurgery, with larger lesions having a negative factor that benefit from osseous grafting of the surgical site following endodontic microsurgery^17^. Moreover, a wide selection of available osseous graft are available and have been advocated ranging from allografts, to xenografts and synthetic materials.

Synthetic graft materials have benefits over available allograft and xenograft materials related to lower cost and have demonstrated comparable clinical results. One of those materials is Bond Apatite^®^ (Augma Biomaterials Int.) a biphasic calcium sulfate utilized in bone surgery in terms of its physicochemical properties, consisting of fine-grained crystals of biphasic calcium sulfate and macro-grains of synthetic hydroxyapatite. Biphasic calcium sulfate is a biocompatible, osteoconductive, bioactive, and bacteriostatic material with a long history in osseous grafting orthopedically and dentally. Those two biomaterials with a similar chemical composition with first use of biphasic calcium sulfate dates back to 1892^18^. This material after mixing sets hard allowing tenting of the area when needed and is fully replaced with host bone over time. This material encourages cells proliferation and angiogenesis while preventing infiltration of epithelial-connective cells into the grafted area eliminating the need for an overlying membrane.

However, the innovation of Bond Apatite is a simple technique for its use. The material is contained in a disposable sterile syringe with a capacity of 1 cm. After activation of the syringes plunger, physiological saline solution is introduced into the solid components within the syringe to activate the material. After such activation, a strong chemical reaction takes place between the saline and calcium sulfate within a few minutes, hydrating the material. The resulting reaction causes the material to take a consistency of hardening cement, without the release of thermal energy permitting the practitioner to place it into the desired site prior to its setting. A barrier membrane is not required in most clinical circumstances, simplifying the procedure, that also reduces the risk of inflammatory complications related to the exposure of the augmentation after suture separation or soft tissue shrinkage. The manufacturer states a gap of up to 5 mm between the flap edges, which is important, for example, in ‘socket preservation’ procedures, does not require membrane usage and can be exposed to the oral environment and soft tissue will migrate to cover the material over a short period of time^19–21^. A case discussing the one-step treatment of an ossifying fibroma of the anterior part of the mandible following endodontic microsurgery with associated retrograde fill of the apex, followed by grafting with Bond Apatite was used in the regeneration of the osseous defect related to the lesion and resulting surgery.

## Case report

**Figure 1:**
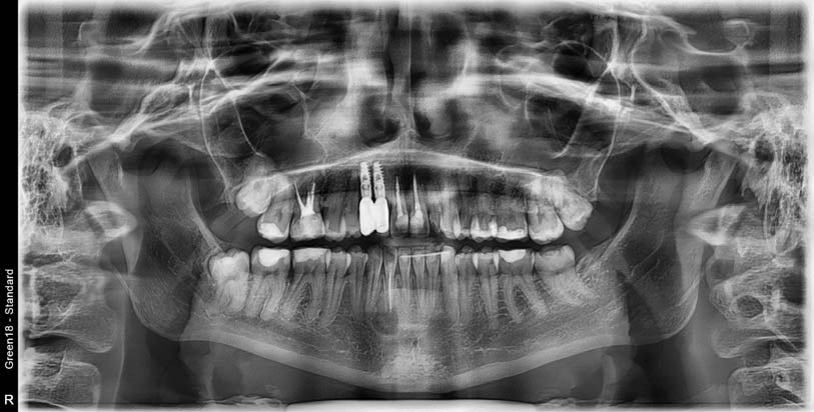
Panoramic radiograph with a lesion associated with the mandibular right lateral incisor.

**Figure 2:**
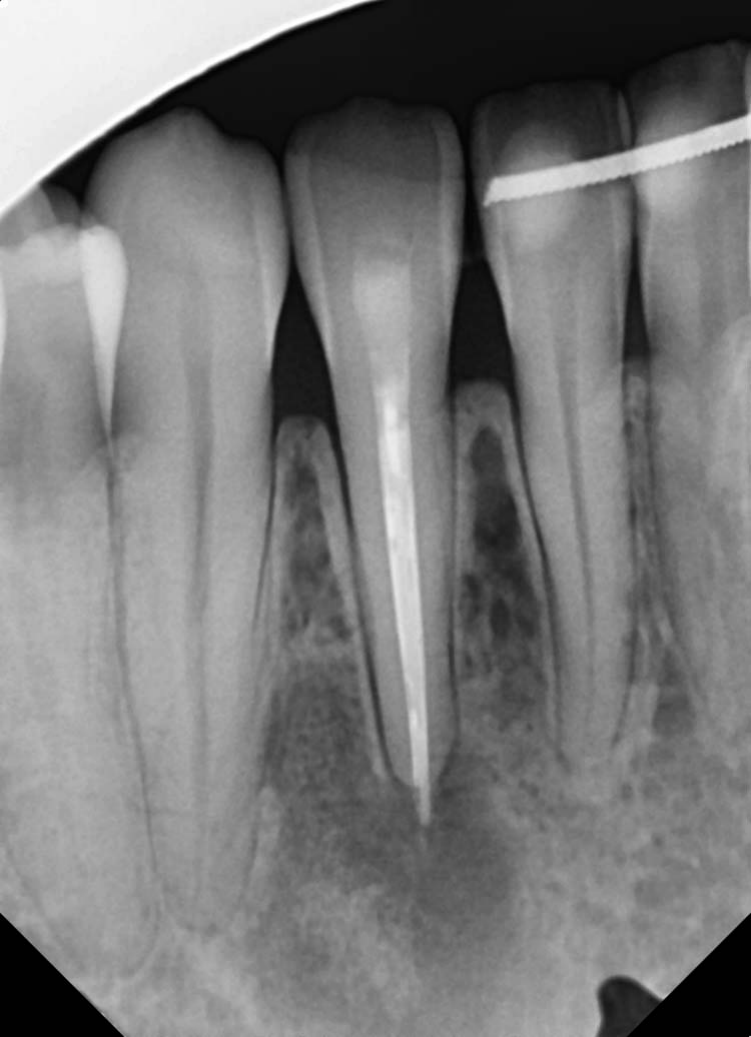
Periapical radiograph demonstrating a large lesion associated with a previously endodontically treated mandibular right lateral incisor.

**Figure 3:**
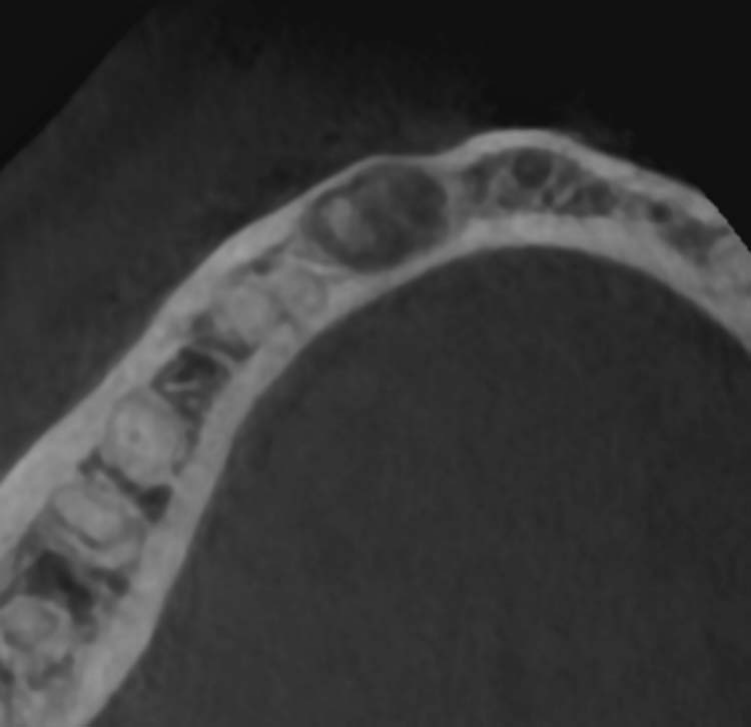
CBCT horizontal view of the mandible demonstrating a mixed cyst in the anterior right area with expansion of the facial plate.

**Figure 4:**
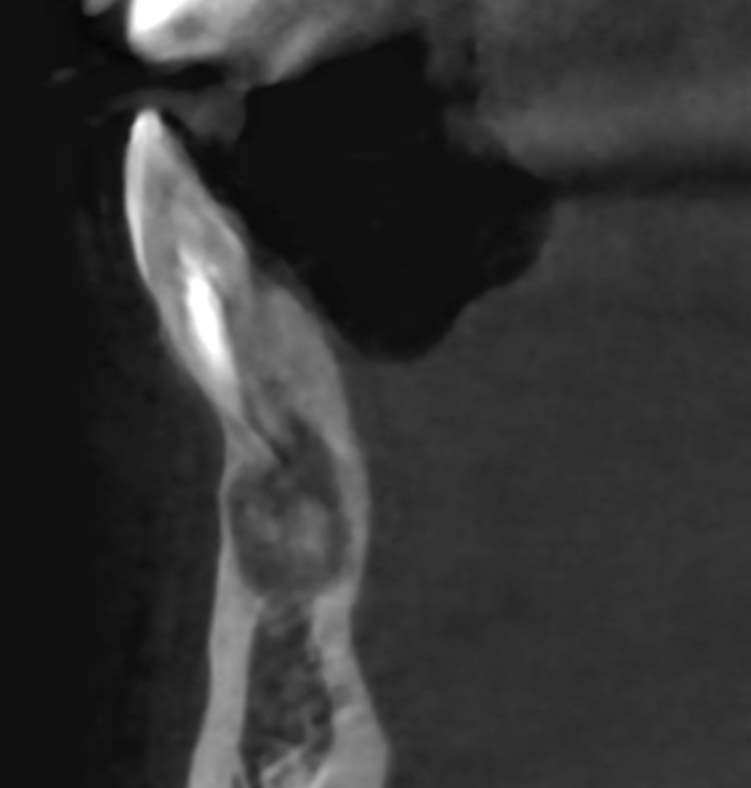
CBCT cross-sectional view at the right lateral incisor demonstrating a mixed lesion with expansion of the area.

**Figure 5:**
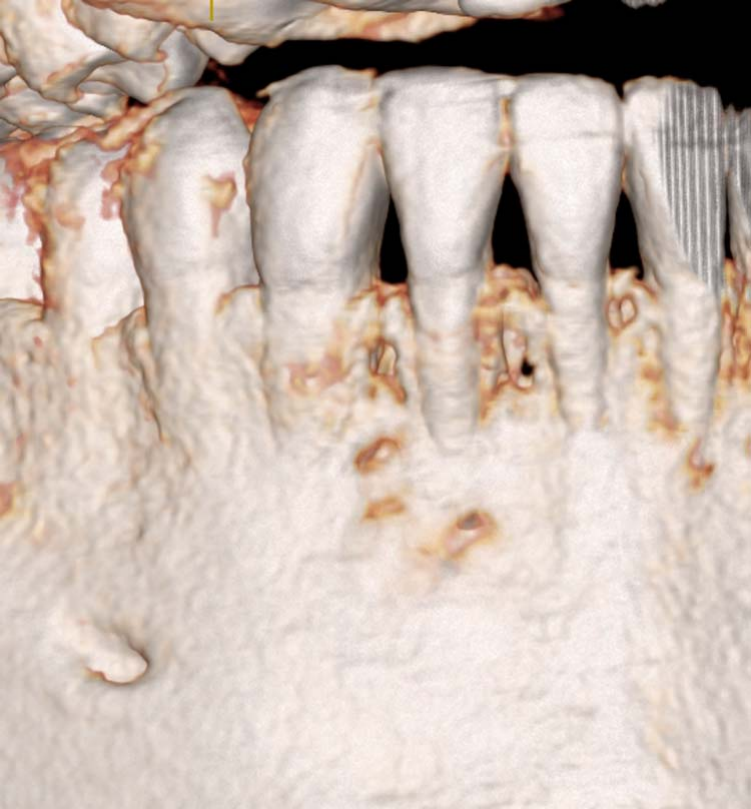
CBCT 3-D reconstruction with the facial plate essentially intact over the identified lesion with some minor perforations of the cortical lamina.

A 26-year-old female patient, presented with pain in the lower right mandibular anterior area on the lateral incisor. No caries was noted via clinical examination or evidence of periodontal disease and pulpal inflammation (pulpitis) was suspected. The patient reported no prior history of inflammatory symptoms and several months prior endodontic treatment had been performed on the right mandibular lateral incisor. A panoramic radiograph was taken to evaluate the tooth and surrounding area and a large periapical area was noted on the tooth (Fig. 1). A periapical radiograph was taken to further evaluate the tooth with confirmation of a large lesion with a mixed area within the cyst and slight over extension of the obturation beyond the apex was noted with no notable external apical resorption of the root (Fig. 2). The lesion appeared like a radicular cyst. In addition, on CBCT scan was performed to evaluate the area three dimensionally. The coronal view noted that the lesion has an expanding character to it (Fig. 3). A cross-sectional view through the tooth, noted the lesion had a irregular shape with a mixed appearance and a radio-opaque area at its center (Fig. 4). A reconstructed view of the facial on the CBCT, noted a relatively intact facial plate overlying the lesion (Fig. 5). Moreover, a small partial damages of external lamina were observed. Based on the clinical findings, the treatment recommended would consist of flap exposure of the facial aspect of the anterior mandible, bone removal to expose the underlying lesion, removal of any pathological tissue making of the lesion, endodontic microsurgery to perform an apicoectomy and retrograde fill, placement of a graft to fill the resulting osseous defect, and flap closure in one-step treatment. Treatment complied with Surgical CAse REport (SCARE) 2000 Criteria^22^.

## Surgical one-step treatment

**Figure 6:**
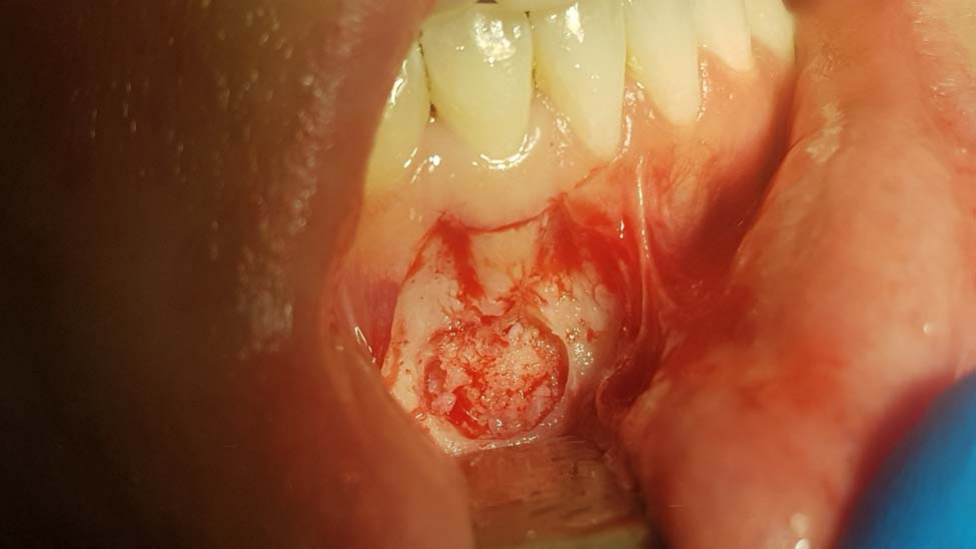
Following flap elevation and trephination of the external lamina of the facial plate, the cyst is visible and is similar to spongiosa bone when touched with an instrument.

**Figure 7:**
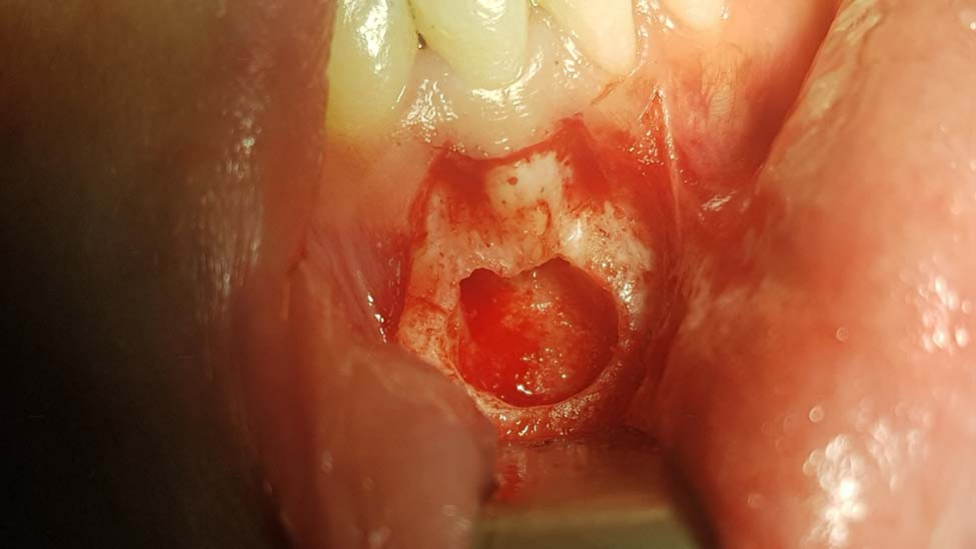
Folowing removal of the cystic tissue with peripheral osseous curretage, resection of the apical portion of the root and placement of retrograde filling in the prepared root the site is ready for ready for graft placement.

Informed consent was reviewed with the patient and the form was signed. Local anesthesia (4% Articaine with epinephrine, Septanest, Septodont) was administered via infiltration into the buccal vestibule. A trapezoidal surgical flap was reflected with vertical releasing incisions between the lateral incisor and canine and also lateral incisor and central incisor with a coronal incision 3 mm below the attached gingival sparing the papillae’s. A full thickness flap was elevated to expose the site. No significant damage was noted upon flap elevation to the facial plates external lamina. A 6 mm wide trephination hole was created over the apical aspect of the tooth to access the underlying lesion (Fig. 6). Upon examination, the tissue under the cortical plate was similar to spongiosa bone but softer than healthy spongiosa bone. The tumor was noticeably demarcated from the surrounding bone. The lesion was curretted to a depth of 1 mm beyond the visible margins of the lesion to remove the pathological tissue and expose the tooth’s apex. The apical extent of the root was removed using a carbide bur in a highspeed handpiece and a retrograde preparation was performed on the root end to a depth of 3 mm. A retrograd fill was performed utilizing MTA (Bio MTA, Cerkamed) to seal the root canal system (Fig. 7).

**Figure 8:**
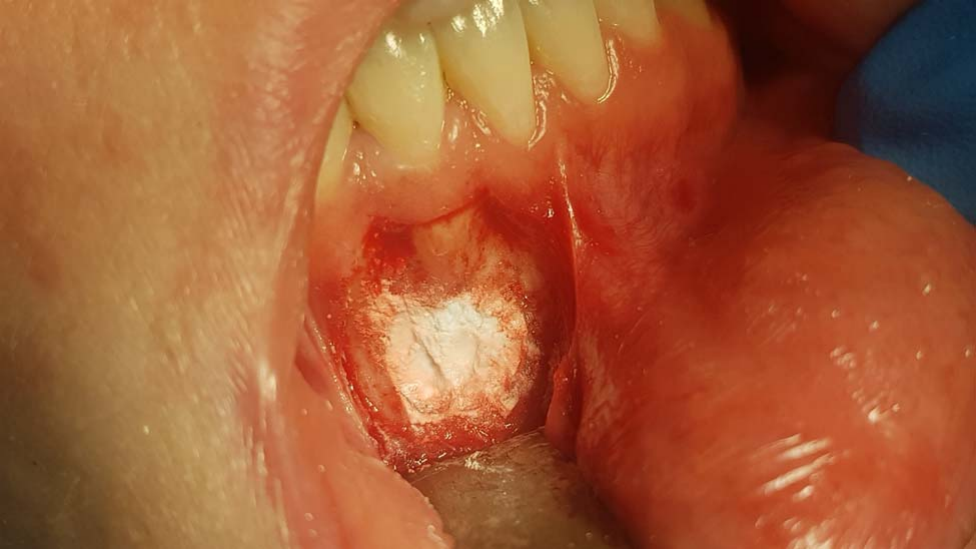
The surgical defect is filled with Bond Apatite.

**Figure 9:**
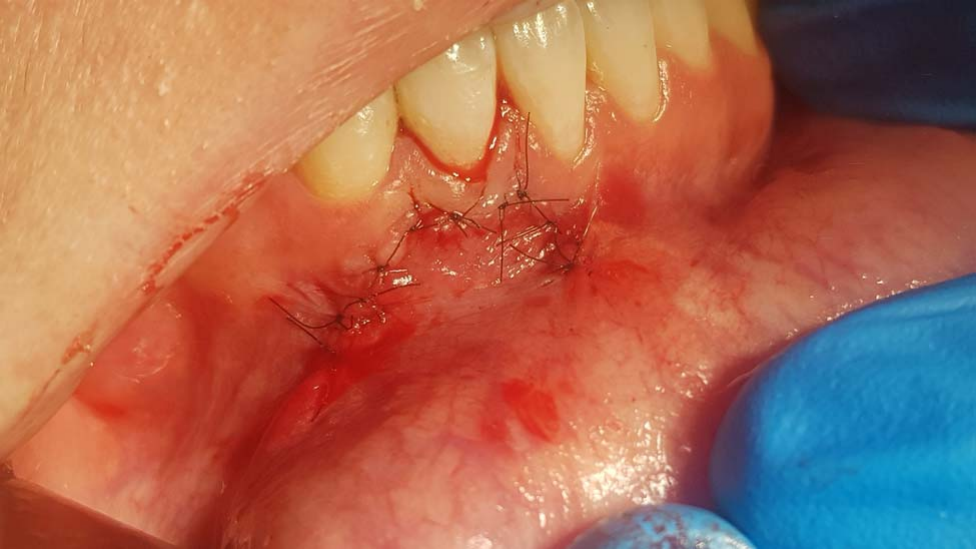
The flap margins are reapproximated and sutures placed to fixate the soft tissue.

The graft material was mixed in the syringe according to the manufacturers instructions and expressed from the syringe into the surgical site to fill the defect and compressed with dry gauze to remove any residual moisture on the material (Fig. 8). The flap was reapproximated to the surrounding tissue and fixated with 5-0 nylon sutures in an interrupted fashion (Fig. 9). The patient was dismissed and scheduled for a postoperative appointment at 7-days. The tissue removed was sent for histological evaluation.

## Histology examination

**Figure 10:**
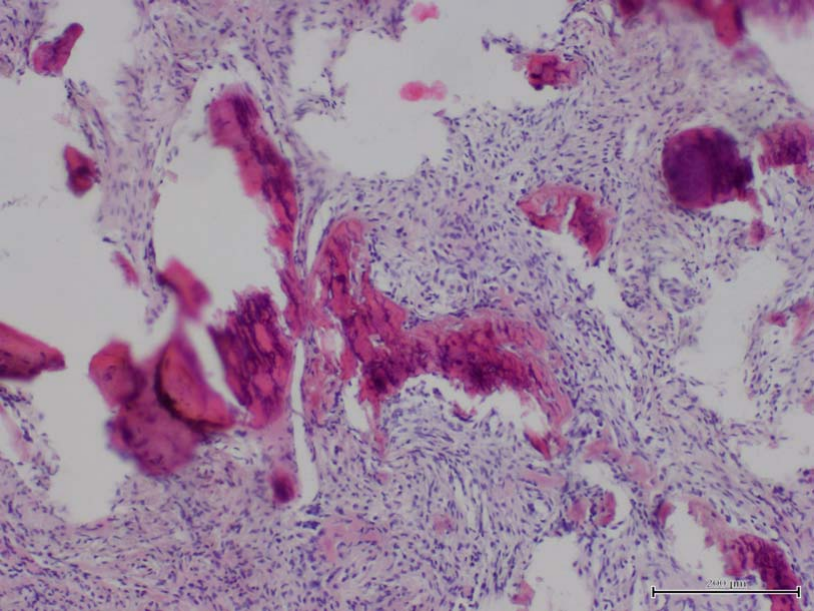
Histological analysis of the cystic tissue that was removed following H&E staining viewed at 100× found connective tissue with fragments of trabecular bone which was identified as an ossifying fibroma.

The operative biopsy was fixed in 10% formalin and placed in a paraffin block. Slicing of the biopsy was performed on a standard microtome (Leica Biosystems, Deer Park). Each 4 mm section was then subjected to a paraffin removal process in a 60°C incubator for 3 h. The samples were then stained with hematoxylin and eosin (standard histochemical procedure) (Fig. 10). Histological analysis confirmed the cyst was an ossifying fibroma.

## Postoperative follow-up

**Figure 11:**
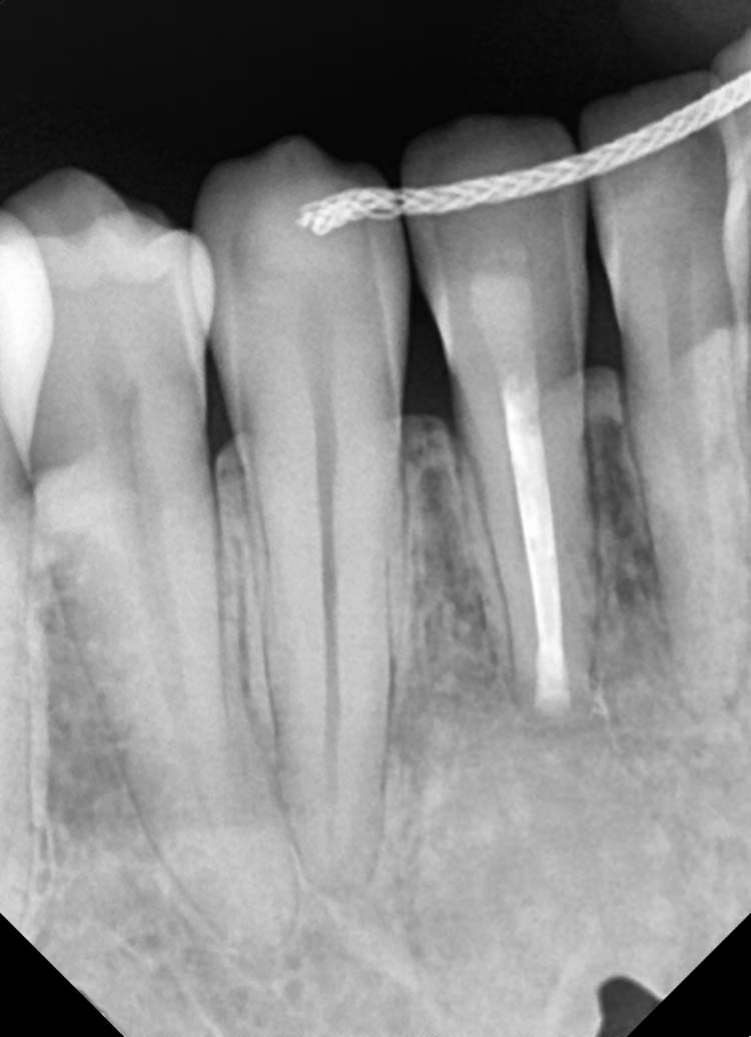
Postoperative periapical radiograph following 7-day healing.

**Figure 12:**
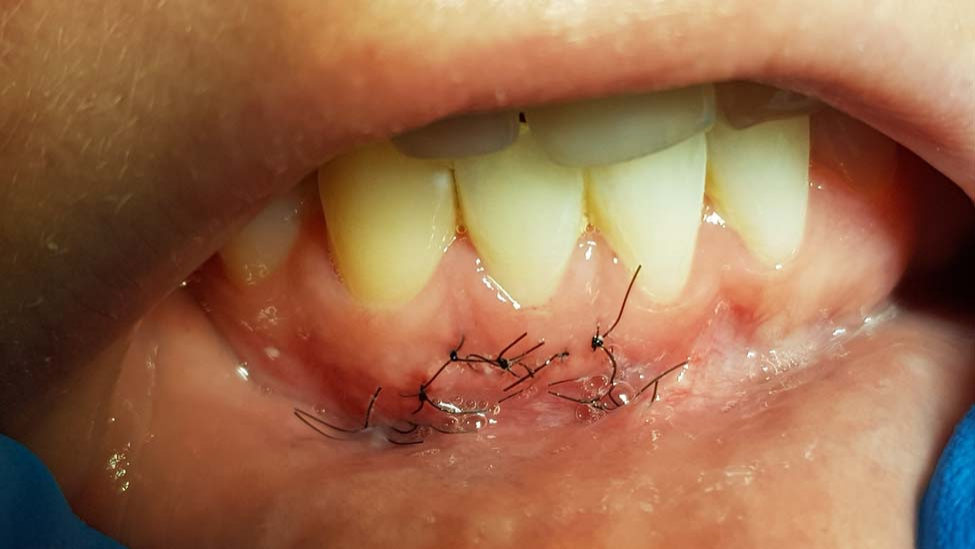
Soft tissue appearance at 7-day postsurgical healing.

**Figure 13:**
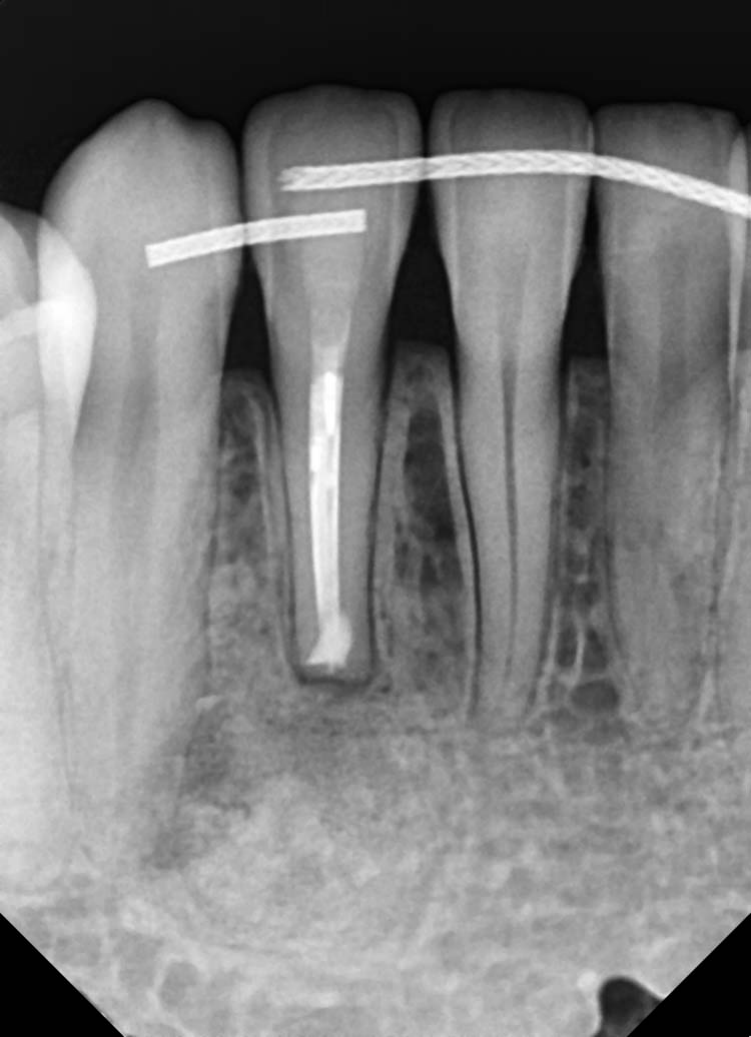
Postoperative periapical radiograph following 6-month healing.

**Figure 14:**
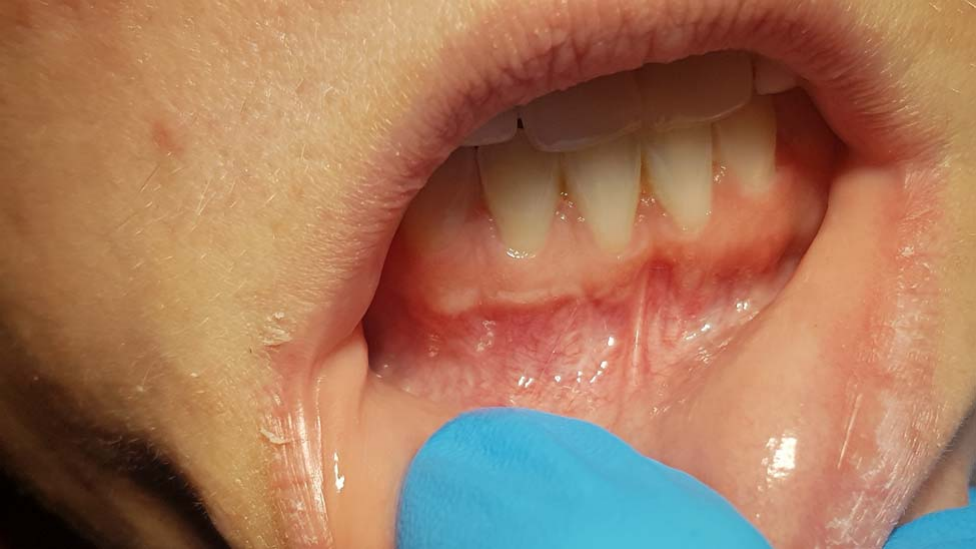
Soft tissue appearance at 6-month postsurgical healing.

**Figure 15:**
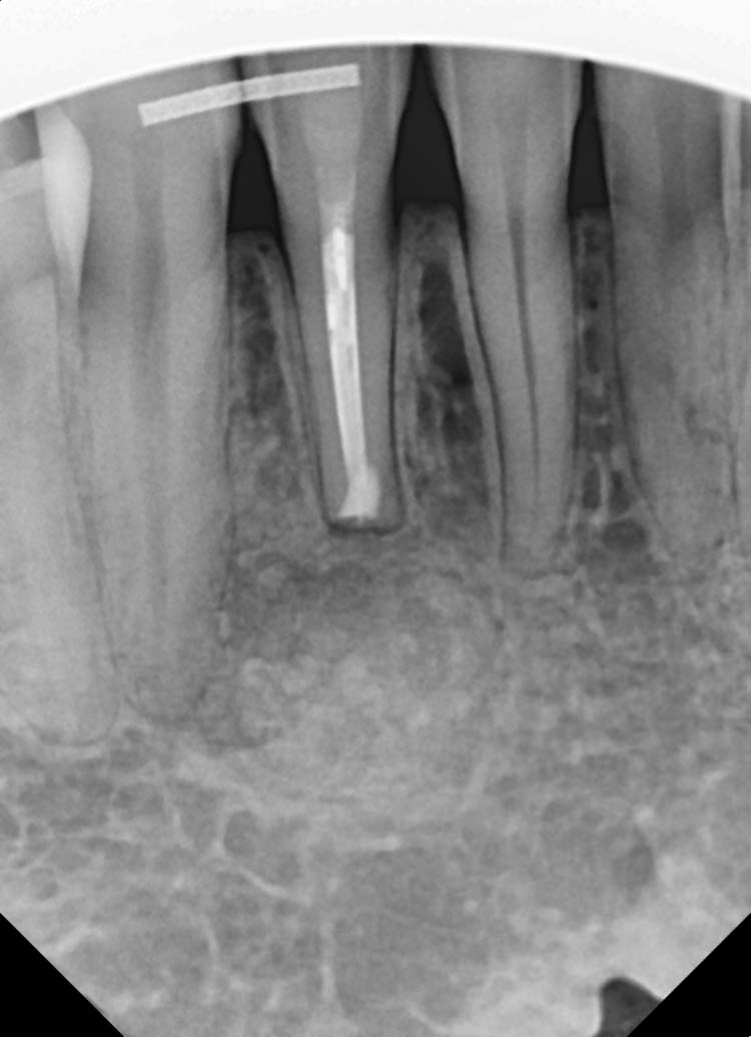
Postoperative periapical radiograph following 12-month healing.

She was presented at the 7-day postsurgical appointment for suture removal and to check general healing. She reported medium pain and discomfort in the first two days following surgery. A control periapical radiograph was taken to document the apical retrograde treatment of the tooth and fill of the lesion with the graft material (Fig. 11). The soft tissue demonstrated no inflammation and the incisions appeared to be closed related to the initial healing (Fig. 12). The patient returned at 6 months for a follow-up and to check radiographically the osseous graft’s healing. A periapical radiograph was taken and good bone remodeling was observed with the grafted area appearing similar in appearance radiographically to the surrounding host bone with a faint border at the periphery between the host bone and the area that had been grafted (Fig. 13). The clinical appearance of the soft tissue appeared normal and no evidence was noted indicating surgery had been performed in the area (Fig. 14). No inflammatory symptoms were observed and the patient reported no clinical symptoms with sensitivity in the area or on the tooth. At a 12-month follow-up a periapical radiograph was taken and complete blending of the grafted area and surrounding host bone was noted with an absence of any pathology (Fig. 15). The patient reported no issues with the tooth or area and it had been absent of any sensitivity since suture removal at 1-week postsurgical treatment.

### Clinical discussion

Apical lesions are a frequent occurrence related to pulpal issues necessitating endodontic treatment. These when limited in size often resolve following endodontic treatment to eliminate the pulpal pathology. But may persist related to inflammatory tissue and infection external to the tooth’s canal system and may require additional treatment to remove that pathological tissue and allow healing. As outlined, other pathological processes may present leading to significant lesion formation that are verified following acquiring a histological sample of the lesion. Evidence has been presented that ossifying fibroma’s may originate in the periodontal ligament or alveolar muscosa^23,24^.

The biopsy sample was analyzed and an ossifying fibroma was histologically identified that is dominated by connective tissue containing cell rich areas with a few fragments of fibrosis. Moreover, in the connective tissue numerous small fragments of spongy and compact bone with areas of partial necrosis present and a significant number of inflammatory cells are observed. Surgical removal of the cyst with thorough curettage of the osseous walls and grafting of the defect provides predictable healing and the desired clinical results sought. Complete surgical excision of the ossifying fibroma is the only effective treatment providing satisfactory results and can be considered a definitive treatment modality^25,26^.

Although the resulting defect following surgical enucleation will heal without the placement of osseous graft material, when a significant sized defect is present graft placement may be advisable. This prevents collapse of the buccal aspect of the ridge during the healing phase while accelerating osseous fill of the defect and conversion to host bone compared to allowing a clot to organize. Various osseous graft materials have been advocated and supported in the literature including allografts and xenograft materials^27^. Those materials require the use of a resorbable membrane over the exterior before repositioning the flap to prevent soft tissue ingrowth into the placed graft material that would compromise the volume when healing of the site is completed. Reports have demonstrated conversion of the materials in the biphasic calcium sulfate to host bone with post healing site biopsies demonstrating over time (6 months) a few particles may remain of the hydroxyapatite granules but are encased in host bone^19–21^.

## Conclusion

The case report presented, as well as the authors experience mimics the literature on Bond Apatite in its use as an osseous graft material and is an effective method for the repair of osseous defects that result from the removal of tumors and cysts of the maxilla and mandible. Treatment of an ossifying fibroma is an ideal application of the use of this biphasic calcium sulfate material allowing tenting of the surgical site over the defect created after cyst removal without the need for resorbable collagen membranes. This simplifies its use and decreases material costs that may hamper patient acceptance of treatment without a decrease in expected clinical results. Furthermore, following soft tissue flap elevation and manipulation is not necessary to get complete primary wound closure without compromise to the expected clinical results. Biphasic Calcium Sulfate is comparable with other osseous graft materials, at the 6 month follow-up, a high clinical effectiveness was observed.

## Ethical approval

This was not a research study, but the case was treated in private practice and the authors are reporting the case to treatment completion.

## Informed consent

Written informed consent was obtained from the patient for publication of this case report and accompanying images. A copy of the written consent is available for review by the Editor-in-Chief of this journal on request.

## Sources of funding

No funding was received for this.

## Author contribution

D.D.: treated and documented the case; O.W.: did the histological analysis; E.R.-W.: reviewed and edited the draft; G.M.K.: wrote the draft and did the literature search.

## Conflicts of interest disclosure

No conflict of interests by any of the authors.

## Guarantor

Dr Kurtzman is corresponding author.

## Research registration unique identifying number (UIN)

Not applicable as this is a case report of a single clinical treatment performed in a clinical dental practice and follow-up not a research study.

## Data availability statement

Not applicable.

## Provenance and peer review

Not commissioned, externally peer-reviewed.
